# ^1^H, ^15^N and ^13^C backbone chemical shift assignment of titin domains A59–A60 and A60 alone

**DOI:** 10.1007/s12104-013-9532-0

**Published:** 2014-01-28

**Authors:** András Czajlik, Gary S. Thompson, Ghulam N. Khan, Arnout P. Kalverda, Steve W. Homans, John Trinick

**Affiliations:** 1 Faculty of Information Technology, Pázmány Péter Catholic University, Budapest, 1083 Hungary; 2 School for Molecular and Cellular Biology and Astbury Centre for Structural Molecular Biology, University of Leeds, Leeds, LS2 9JT UK; 3 Executive Office, Newcastle University, King’s Gate, Newcastle upon Tyne, NE1 7RU UK

**Keywords:** Muscle protein, Titin A-band, Large super-repeat unit, Fibronectin type III domain tandem

## Abstract

The giant protein titin is the third most abundant protein of vertebrate striated muscle. The titin molecule is >1 μm long and spans half the sarcomere, from the Z-disk to the M-line, and has important roles in sarcomere assembly, elasticity and intracellular signaling. In the A-band of the sarcomere titin is attached to the thick filaments and mainly consists immunoglobulin-like and fibronectin type III-like domains. These are mostly arranged in long-range patterns or ‘super-repeats’. The large super-repeats each contain 11 domains and are repeated 11 times, thus forming nearly half the titin molecule. Through interactions with myosin and C-protein, they are involved in thick filament assembly. The importance of titin in muscle assembly is highlighted by the effect of mutations in the A-band portion, which are the commonest cause of dilated cardiomyopathy, affecting ~1 in 250 (Herman et al. in N Engl J Med 366:619–628, 2012). Here we report backbone ^15^N, ^13^C and ^1^H chemical shift and ^13^Cβ assignments for the A59–A60 domain tandem from the titin A59–A69 large super-repeat, completed using triple resonance NMR. Since, some regions of the backbone remained unassigned in A60 domain of the complete A59–A60 tandem, a construct containing a single A60 domain, A60sd, was also studied using the same methods. Considerably improved assignment coverage was achieved using A60sd due to its lower mass and improved molecular tumbling rate; these assignments also allowed the analysis of inter-domain interactions using chemical shift mapping against A59–A60.

## Biological and medical context

Titin, a major component of vertebrate striated muscle sarcomeres, is the largest known polypeptide, with isoforms up to nearly 4 MDa (Tskhovrebova and Trinick 2003). This is mostly folded into a string of about 300 immunoglobulin (Ig) and fibronectin (Fn, type III)-like domains, both of which have β-sandwich folds of 7–8 strands and about 100 amino acids. The filamentous molecule spans from the Z-discs at the ends of the sarcomere (N-terminus) to the M-line in its middle (C-terminus), a distance of ~1 μm. In the A-band titin is integral with thick filaments and is thought to control exact filament assembly (Whiting et al. 1989) via interactions with myosin, C-protein and other filament components. In the C-zone of the A-band the Ig and Fn domains are arranged in repeating patterns called large super-repeats: Ig–Fn–Fn–Fn–Ig–Fn–Fn–Fn–Ig–Fn–Fn (Labeit et al. 1992). Thus domains at the same positions in different super-repeats are most similar. This pattern is repeated 11 times, making 121 domains in total and forming nearly half the molecule. Next generation sequencing has recently shown that A-band titin is of great importance in heart disease. Mutations throughout this region are the commonest cause of the important disease dilated cardiomyopathy, which affects 1 in 250 in the population and is a major cause of death (Herman et al. 2012).

Our long-term aim is to determine the structure and dynamical properties of the A59–A69 large super-repeat unit using overlapping two and three domain constructs (Tskhovrebova et al. 2010; Czajlik et al. 2012). By inference, this should allow modeling of the entire large super-repeat region spanning 0.5 μm. Here we report chemical shift assignments of the backbones of the A59–A60 fibronectin type III (Fn3) double and A60sd single domain constructs using triple resonance NMR. Assignments of backbone resonances in these molecules provide a basis for understanding inter-domain flexibility, domain-domain interactions and the formation of higher oligomer in the system, based on homology models and chemical shift based backbone folds using residual dipolar couplings, paramagnetic relaxation enhancements, ^1^H–^15^N relaxation data and chemical shift perturbations, without the need for full sidechain assignment.

## Methods and experiments

The A59–A60 domain tandem and A60sd single domain were cloned into the pET 15b_A170 and PET19b vectors, respectively. They were over-expressed as a N-terminal His6-tagged fusion protein in* Escherichia coli* strain BL21(DE3). A59–A60 was uniformly ^15^N, ^13^C and ^2^H-labeled and A60sd was uniformly enriched in ^15^N and ^13^C. Cells were grown at 37 °C in D_2_O-M9 minimal medium containing ^15^NH_4_Cl (1 g/L) and either [100 %-D7 and 100 %-^13^C] glucose (4 g/L) or [100 %-^13^C] glucose (4 g/L) as the sole nitrogen and carbon sources. For induction of expression, 1 mM IPTG was added when the cells reached an OD_600_ of 0.7, after which they were left shaking at 37 °C for an additional 15 h. The cells were then disrupted by sonication in a 50 mM TRIS buffer (pH 7.9) containing 6 M guanidinium chloride, 0.5 M NaCl and 15 mM DTT and harvested by centrifugation. Finally the proteins were purified from the supernatant using Ni–NTA resin and eluted with 50 mM EDTA (pH 6.2). Typically 5 mg protein was purified from 1 l of A59–A60 culture. For A60sd yields were much higher, ~15 mg/l of culture. Purity and isotope enrichment were checked by SDS-PAGE and mass spectrometry.

NMR samples of both purified proteins (~1 mM) were prepared in 0.5 M NaCl, 50 mM MES, 10 mM DTT and 0.01 % azide, pH 6.5. Data were recorded at 293 K on Varian INOVA 600 and 750 MHz spectrometers equipped with ^1^H,^13^C,^15^N triple resonance probes with z-axis pulsed field gradients; the 750 MHz spectrometer was equipped with a cryogenic probe. Sequence specific backbone assignments were obtained from 2D ^15^N HSQC-TROSY, 3D HNCA, HN(CO)CA, HN(CA)CB, HN(COCA)CB, HNCO and HN(CA)CO spectra. Data was processed using NMRPipe (Delaglio et al. 1995) and spectra were analyzed using CCPNMR analysis 1.0 (Vranken et al. 2005). Chemical shift referencing was carried out using DSS (2,2-dimethyl-2-sila-pentane-5-sulfonic acid). Measurements of {^1^H}–^15^N heteronuclear nOes Heteronuclear (Farrow et al. 1994) were used at a ^15^N frequency of 60.78 MHz with a proton saturation sequence using a 120° pulse applied every 5 ms over 3 s during the 5-s relaxation delay. Values for the {^1^H}–^15^N nOes were determined from the ratio of peak intensities with and without proton saturation. 1D TRACT experiments (Lee et al. 2006) were acquired and relaxation rates for the α and β spin states averaged over the amide spectrum were analysed by an in-house python script to give a lower limit for the rotational correlation time.

## Assignments and data deposition

**Fig. 1:**
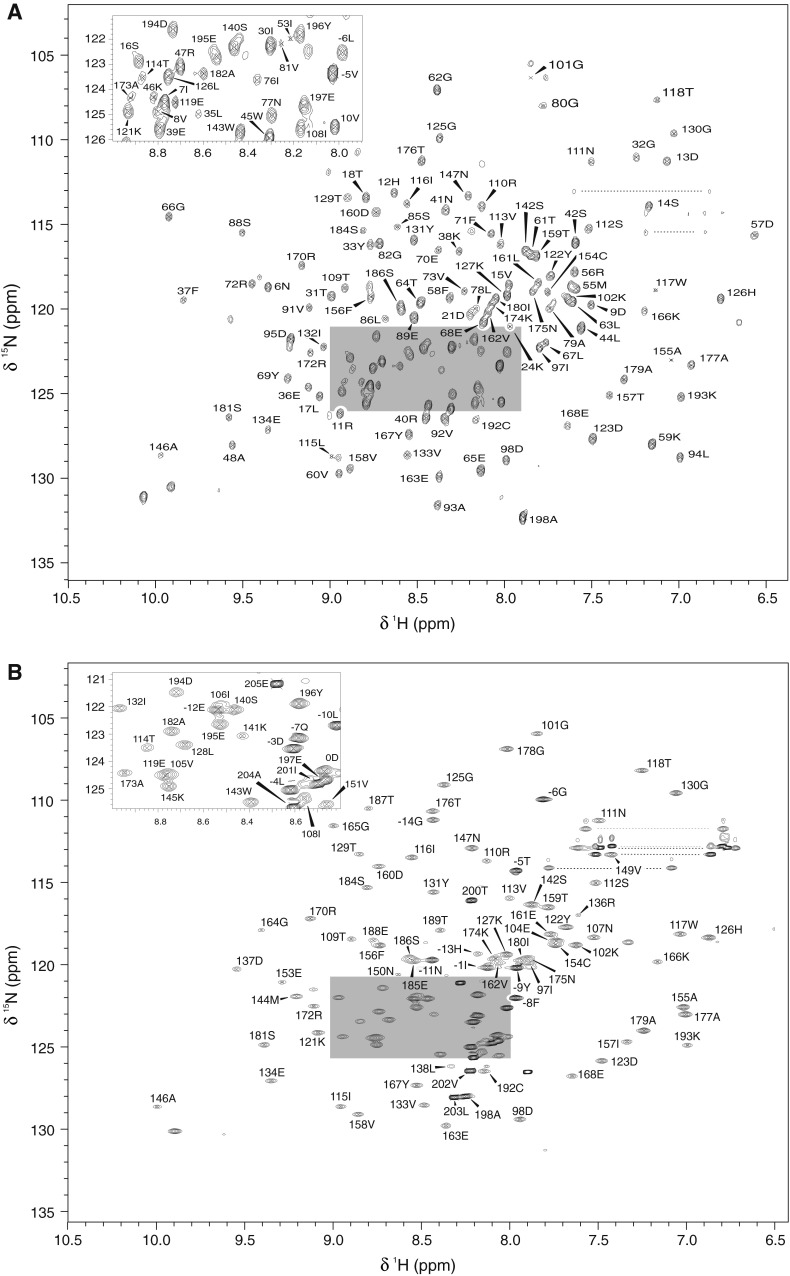
**A** Two-dimensional ^1^H–^15^N HSQC-TROSY spectrum of A59–A60 tandem at 750-MHz ^1^H frequency. **B** Two-dimensional ^1^H–^15^N HSQC-TROSY spectrum of A60sd at 750-MHz ^1^H frequency. Data were acquired at 293 K using a uniformly ^15^N, ^13^C, (^2^D for A59–A60) labelled protein sample (~1 mM) dissolved in 0.5 M NaCl, 50 mM MES, 10 mM DTT (pH 6.5). Note all N-terminal tag residues are labeled with negative numbers, while residues in A60sd and A59–A60 are labeled as consecutive positive numbers from the start of the A59 sequence

Figure 1A shows the ^15^N–^1^H HSQC-TROSY spectrum of A59–A60 illustrating the quality of the data and breadth of assignment. The resonances are well dispersed indicating the folded state of the protein. Excluding the resonances of N-terminal His6-tag and 4 Pro amino acids from the very beginning of the domain (labelled −24 to 0), backbone assignment of A59 from A59 to A60 proved nearly complete [90.5 % (76/84) ^1^HN/^15^N, 95.5 % (86/90) ^13^Cα, 87.9 % (73/83) ^13^Cβ, 88.9 % (80/90) ^13^C’]. In contrast, assignment of A60 in A59–A60 was not as exhaustive: 80.0 % (76/95) for ^1^HN/^15^N, 86.4 % (89/103) for ^13^Cα, 67.7 % (65/96) for ^13^Cβ, and 73.8 % (76/103) for ^13^C’. Furthermore, while in A59 most of the unassigned resonances were found in loop regions, in A60 resonances from three spatially separate regions from the first, third and seventh β-strands were totally missing (103Pro, 104Glu, 105Val; 135Lys, 136Arg, 137Asp and 187Thr, 188Glu, 189Thr, 190Ile). In order to increase the assignment rate and to help study the interaction between the domains, A60 was expressed alone (A60sd). The completeness of the assignments for A60sd [excluding the N-terminal His6-tag and trailing N terminal residues (25 residues labelled −24 to 0) and the C-terminal trailing residues (residues labelled 199–205)] proved much better than for A59–A60 (Fig. 1B) with coverage of: 94.7 % (90/95) for ^1^HN/^15^N, 98.1 % (101/103) for ^13^Cα, 72.9 % (70/96) for ^13^Cβ, and 93.2 % (96/103) for ^13^C’. Thus, the source of the missing assignments in the A59–A60 may reasonably be attributed to T2 broadening due to local chemical exchange and the correlation time of the full tandem.

**Fig. 2:**
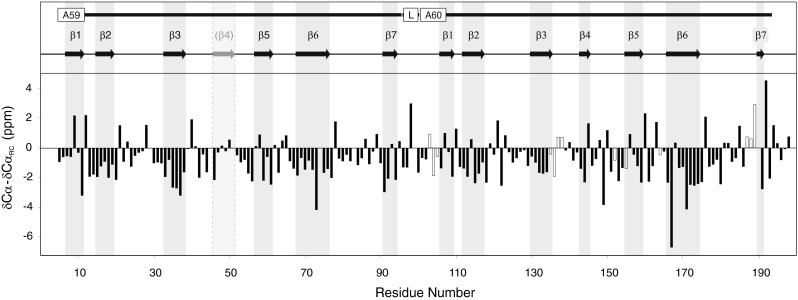
^13^Cα chemical shift deviations of the A60 single domain compared to sequence dependent random coil values (ΔδCα−δCα_RC_ = δobserved−δrandom coil; random coil values were based on those from Wishart et al. 1995). For those shifts where no assignments were available for the A59–A60 tandem those from A60sd were used (*open bars*). Regions identified as being in a β-sheet conformation based on CSI and TALOS+ are indicated by a* grey ground*. No α-helical secondary structure was identified. The position of the putative β-sheet A59-β4, which is predicted by PSIPRED, and the canonical Fn3 fold are shown with a dashed border

Consensus analysis of secondary structure based on CSI (Wishart and Sykes 1994) and TALOS+ (Shen et al. 2009), indicate the presence of thirteen β-strand regions (Fig. 2): A59-β1 (7–11), A59-β2 (15–19), A59-β3 (33–38), A59-β5 (57–61), A59-β6 (68–76), A59-β7(91–94), A60-β1 (106–109), A60-β2 (112–117), A60-β3 (130–135), A60-β4 (143–145), A60-β5 (155–159), A60-β6 (166–175), A60-β7 (190–191). The secondary structure elements observed are in good agreement with those for two Fn3 domains, with the exception that the position of A59-β4 could not be clearly identified from the observed chemical shifts. Interestingly the very short β-sheet observed for A60-β7 is not consistent with the secondary structure of the β7 strand from the NMR of the domain A71 (Goll et al. 1998, 1BPV) which is situated at the same position in the next super-repeat unit as the A60. However, it is completely consistent with the sequence dependant secondary structure predicted by PSIPRED (Buchan et al. 2010; Jones 1999).

**Fig. 3:**
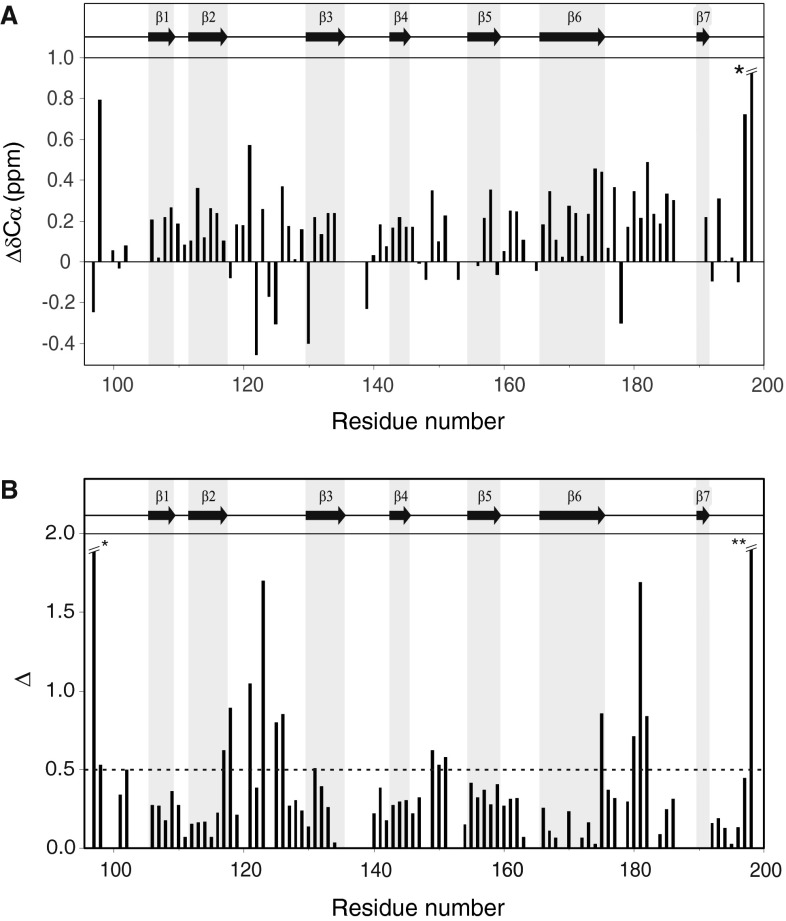
**A** Cα chemical shift differences between the A60 domain of A59–A60 and A60sd For clarity the data for residue 198 (Δppm Cα = 3.26 ppm, labelled *) was truncated on the *y axis*. **B** Bottom, a graph of the shift metric Δ for all residues in A60 domain. Δ = [(δ^15^N)^2^ + (5 × δ^1^H)^2^]^0.5^ where δ^15^N and δ^1^H are the chemical shift differences between the A60 domain of A59–A60 and A60sd. Regions identified as being in a β-sheet conformation using CSI and TALOS+ are indicated by a* grey ground*, the cutoff for significant values of Δ > 0.5 ppm (shown by a *dashed line*). Residues 97 and 198, labeled * and ** respectively, have Δ values of 2.28 and 4.53

In order to prove that the secondary structure of the C-terminal domain of A59–A60 and A60sd are the same, their Cα chemical shifts were compared. The Cα chemical shifts for A60 from the A59–A60 tandem and A60sd were highly correlated (R^2^ = 0.99) and, excluding changes at the termini where different flanking residues were present, only small shift changes were observed (Fig. 3A), with the largest being 0.57 ppm indicating very little change in overall secondary structure. However, consistent differences in chemical shifts (δCα ≥ 0.3 ppm) are observed for the loops regions between β-strands A60-β2 and A60-β3, as well as between β-strands A60-β6 and A60-β7. This suggests that there is an inter-domain interaction. In order to examine the interaction between A59 and A60 further, the changes in chemical shift between the ^1^H–^15^N HSQC spectra of the C-terminal domain of A59–A60 and A60sd were calculated (Fig. 3B) using the chemical shift distance metric Δ = [(δ15 N)^2^ + (5 × δ1H)^2^]^0.5^. Aside from the termini, only the loop regions between β-strands A60-β2 and A60-β3, A60-β4 and A60-β5, as well as a number of residues in β- sheets A60-β6 and β7, show a Δ > 0.5 ppm, indicating that their environment has changed significantly. As these regions are localized to the N-terminal face of A60 (assuming a Fn3 fold) this suggests that there are distinct interactions between A59 and A60. Three further strands of evidence support a lack of flexibility between the two domains. Firstly from the observed secondary structure the linker between the β-sheets A59-β6 and A60-β1 is extremely short (residues 95–100). Secondly the observed correlation time of 21 ns for A59–A60 which was measured using a TRACT experiment is consistent with that expected for a rigid double domain construct with small amounts of a higher oligomer present (Tskhovrebova and Trinick 2003). Finally {^1^H}–^15^N heteronuclear nOe measurements show that the linker region has nOes intensities covering a range from 0.77 ± 0.03 to 0.78 ± 0.03, which are in agreement with the observed nOes for the secondary structure elements in the same system which have an average value of 0.76 ± 0.05 over secondary structure elements. Overall these results clearly point to the conclusion that the A59–A60 double domain has an inflexible linker and a structured domain–domain interface. This observation therefore has important implications for the overall flexibility, structure and biological role of the large super repeat in titin and its role in myosin assembly.

Backbone ^1^H, ^13^C and ^15^N resonance assignments of titin A59–A60 tandem and A60sd single domain have been submitted to the BMRB (http://www.bmrb.wisc.edu) under accession numbers 19010 and 19011, respectively.
